# The use of the buccal fat pad flap for oral reconstruction

**DOI:** 10.1186/s40902-017-0105-5

**Published:** 2017-02-25

**Authors:** Min-Keun Kim, Wonil Han, Seong-Gon Kim

**Affiliations:** 1 Department of Oral and Maxillofacial Surgery, College of Dentistry, Gangneung-Wonju National University, 7 Jukhyun-gil, Gangneung, 25457 Republic of Korea; 2 Han Dental Clinic, Guri, Republic of Korea

**Keywords:** Buccal fat pad flap, Defect, Reconstruction, Oral, Wound epithelialization

## Abstract

Many congenital and acquired defects occur in the maxillofacial area. The buccal fat pad flap (BFP) is a simple and reliable flap for the treatment of many of these defects because of its rich blood supply and location, which is close to the location of various intraoral defects. In this article, we have reviewed BFP and the associated anatomical background, surgical techniques, and clinical applications. The surgical procedure is simple and has shown a high success rate in various clinical applications (approximately 90%), including the closure of oroantral fistula, correction of congenital defect, treatment of jaw bone necrosis, and reconstruction of tumor defects. The control of etiologic factors, size of defect, anatomical location of defect, and general condition of patient could influence the prognosis after grafting. In conclusion, BFP is a reliable flap that can be applied to various clinical situations.

## Introduction

Soft tissue coverage is an essential step for successful wound healing. Intraoral wounds have certain unique features compared to other wound sites. The soft tissue overlying the alveolar bone is relatively thin, and there is no fatty layer in the gingiva. Therefore, vascularized skin graft is too bulky in most cases, and the color of skin graft is not matched to that of the oral mucosa [1]. Free mucosal graft from the palate has a well-matched color and similar thickness to the gingiva [2]. However, the size of the palatal mucosa is limited. As the palatal mucosal graft is a free graft, it is not indicated for poorly vascularized recipient beds [2].

Intraoral soft tissue defect can be induced by various diseases or complications. Cleft palate and cleft alveolus are congenital defects that accompany bone defects [3, 4]. Oroantral fistula is often observed after tooth extraction in cases of severe sinus pneumatization [5, 6]. Tumor or trauma also shows various degrees of soft tissue defect [7, 8]. Recently, many cases of medication-induced osteonecrosis of the jaw have been reported, and these patients have denuded bone surface [9]. Although the size, location, and etiology are different from case to case, the soft tissue defect with problems in wound healing is a common feature.

Vascularized grafts may be considered as first choice of treatment in oral reconstruction, but have limitations. Patients with compromised wounds usually have poorly vascularized tissue, and patients with severe diabetes mellitus have difficulties with capillary regeneration [10]. These patients have demonstrated higher rates of postoperative infection and graft failure [10]. Patients receiving radiation therapy or chemotherapy also experience problems in wound healing [11]. Moreover, patients receiving high doses of bisphosphonate often show avascular jaw bone necrosis following oral surgery [9]. Although revision surgery is attempted for these patients, vascularized grafts are the only conventional method that have not failed [12]. However, vascularized grafts should be performed under general anesthesia and require a long operation time. Donor site morbidity and an additional scar are the disadvantages of using vascularized grafts [12].

Buccal fat pad flap (BFP) has been used for the reconstruction of maxillary defects induced by tumor since it was first reported in 1977 [13]. From then, many clinical applications of BFP have been introduced. The buccal fat pad appears 3 months in utero and continuously grows until birth [14]. There is little change in the volume of buccal fat during aging, and it is approximately 10 mL [14]. Therefore, it is a reliable flap for the reconstruction of oral defects. Most published studies have reported a high success rate among BFP procedures due to BFP’s rich vascularity, proximity to the recipient site, low donor-site morbidity, and simple surgical procedure for grafting [15]. This review discusses the anatomical background and surgical technique of BFP. In addition, the clinical application of BFP and its results are discussed.

## Review

### Anatomical background and surgical technique

#### Anatomic background

The buccal fat pad appears at 3 months in utero and continuously grows until birth [14]. It protrudes at the anterior border of the masseter muscle and extends to the parotid duct, where it rests on the buccopharyngeal fascia, which covers the buccinator muscle [16]. There is little change in the volume of buccal fat during aging, and it is approximately 10 mL [14].

**Fig. 1:**
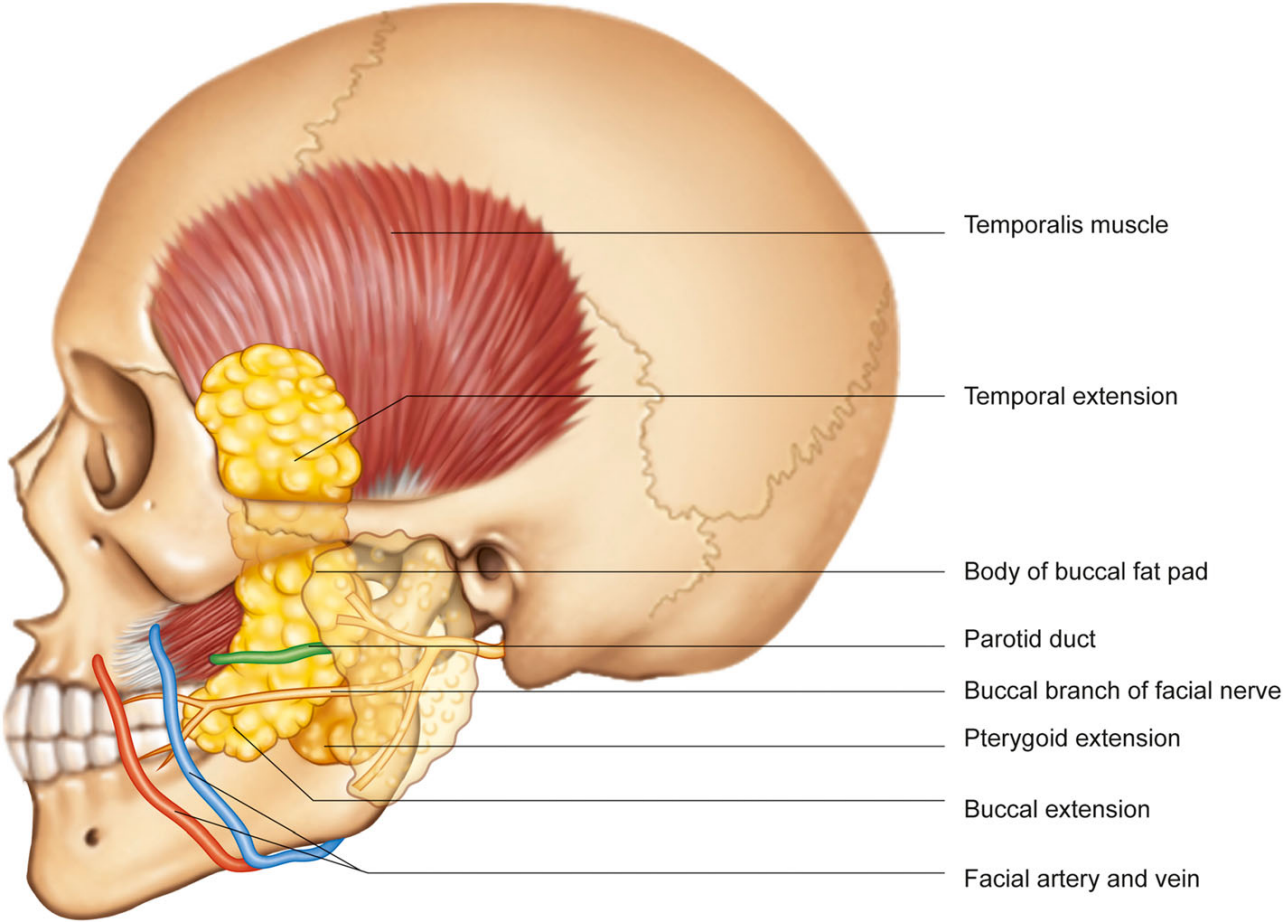
Anatomical location of the buccal fat pad. The buccal fat pad is composed of a main body and four extensions (temporal, buccal, pterygoid, and pterygopalatine)

The buccal fat pad is composed of lobes and highly mobile structures (Fig. 1). It has a main body and four extensions: temporal, buccal, pterygoid, and pterygopalatine [15]. The main body is surrounded by the buccinator muscle, masseter muscle, and zygomatic arch. The main body is positioned along the posterior maxilla and covered with a thin capsule. The parotid duct pierces the buccinator at the anterior border of the buccal fat pad [16]. The average volume of the fat pad is 9.6 mL (range, 8.3–11.9 mL). The average weight of the fat pad is 9.3 g (range, 8–11.5 g). When properly dissected, the buccal fat pad provides a 6 × 5 × 3-cm graft. The average thickness is 6 mm, and this can cover an area of 10 cm^2^ [16, 17].

The buccal fat pad has abundant blood supplies from the maxillary artery and the superficial and deep temporal artery. There are rich capillary networks within the capsules that cover the fat pad. Arterioles enter the capsule from several directions and break up into capillary plexuses. Most of the blood from the fat pad drains into the facial vein [16]. Stensen’s duct is an adjacent anatomic structure, so it is easily encountered when extracting the buccal fat pad. Thus, surgeons should take care not to damage this apparatus.

### Surgical technique

**Fig. 2:**
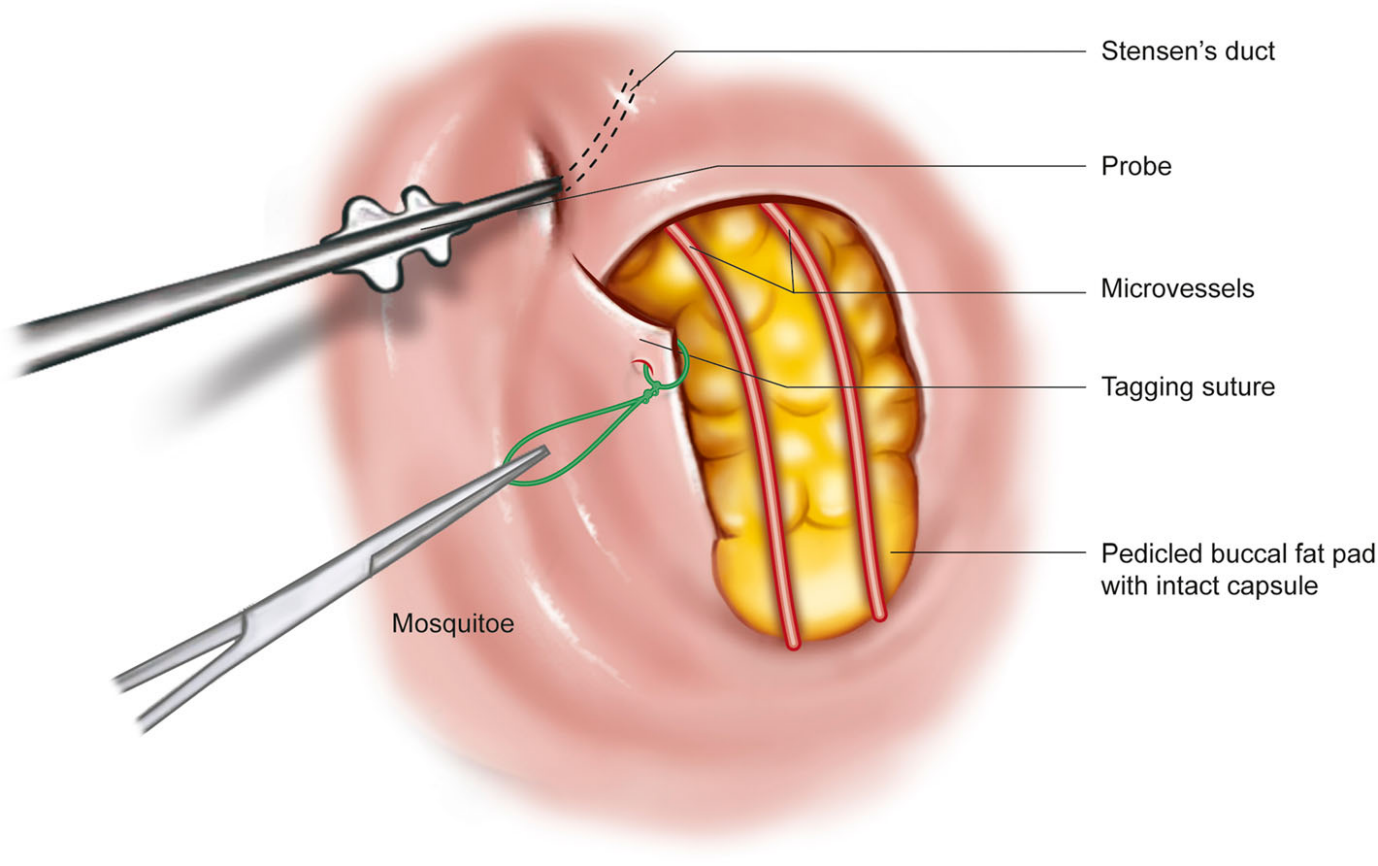
Surgical procedure for the buccal fat pad flap. A blunt dissection is carefully performed without injuring the capsule overlying the fat pad. After the superficial fascia of the face was cut, the fat pad herniated spontaneously

After lidocaine (1%) with 1:100,000 epinephrine is infiltrated, Stensen’s duct should be identified with a lacrimal probe before incision to avoid damaging it during the procedure. A 2–3-cm mucosal incision was made at least 2 cm below the Stensen’s duct. Two or three tagging sutures were placed at the margin of the mucoperiosteal flap to gain appropriate surgical fields. The buccinator and zygomaticus major muscles were cut, and blunt dissection was carefully performed to create sufficient openings for herniating the fat pad without injuring the capsule overlying the fat pad. After the superficial fascia of the face was cut, the fat pad herniated spontaneously (Fig. 2). The capsules overlying the fat pad should not be torn so as to maintain its volume, and the arterioles and venules overlying the fat pad should be preserved to maintain the rich blood supply. Tissue forceps were used for the traction of the fat pad with minimal force to avoid tearing the capsule. Pedicled buccal fat pad was sutured and positioned using absorbable suture materials with minimal tension. Making the incision at the bone is a good technique for maintaining the position of the fat pad.

### Clinical application

#### Oroantral fistula associated with tooth extraction or dental implant removal

Oroantral fistula is the state of patent communication between the oral cavity and the maxillary sinus [5, 18]. Although it is common after a tooth or dental implant extraction, patent opening to the maxillary sinus can also be induced by a pathological condition such as osteonecrosis, cyst, or tumor, or by congenital deformity such as cleft palate. As the extent of bony defect is generally larger in pathological conditions and congenital deformities than in cases requiring a simple extraction, pathological conditions related to oroantral communications are discussed separately.

**Fig. 3:**
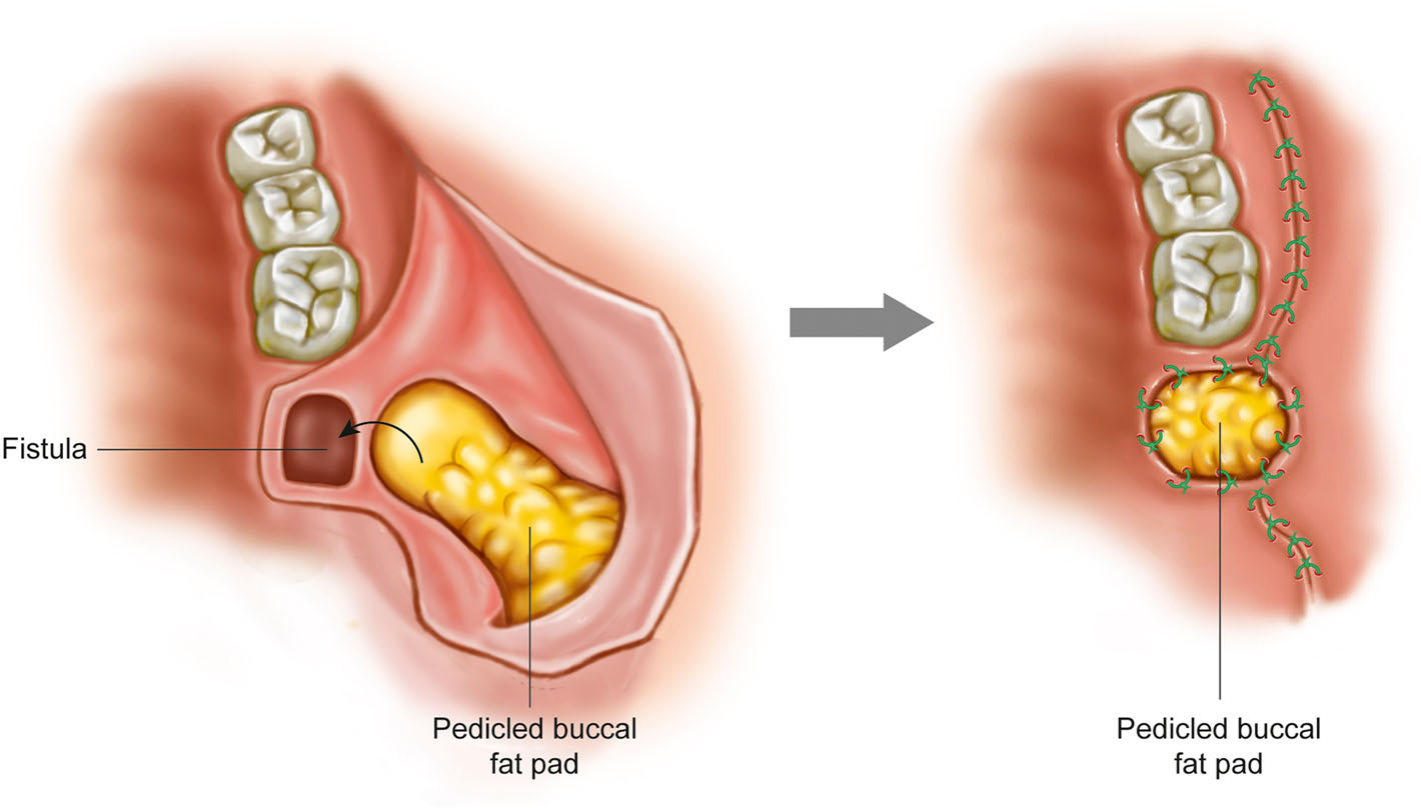
Closure of oroantral fistula by the buccal fat pad flap

Oroantral fistula associated with extraction is mainly observed in the maxillary premolar or molar area [5]. Patients with severe sinus pneumatization are vulnerable to oroantral fistula after extraction [18, 19]. Root fracture and subsequent improper instrumentation is also a cause of oroantral fistula. Oroantral fistula can appear immediately after the removal of a tooth or dental implant and remain unhealed for over 1 month [5]. Small-sized perforations (≤2 mm) can be allowed to heal spontaneously. Persistent communications should be treated because food and fluid regurgitate into the maxillary sinus and may result in sinusitis [20, 21]. The traditional methods for treating oroantral fistula have been buccal advancement flap or rotational palatal flap. Vestibular shallowing is a drawback of the buccal advancement flap [18]. Moreover, patients with damaged gingiva or those who received a previous closure operation cannot be indicated for the buccal advancement flap [18]. However, BFP demonstrated high success rates, even in previously operated cases [19]. The surgical procedure of BFP graft for the treatment of oroantral fistula is very simple (Fig. 3).

Studies involving less than five patients were excluded from the present review. In the literature, all studies cited tooth or dental implant removal as an etiology of oroantral fistula. BFP was the only treatment used in most studies. In one study, two patients received collagen strip as an additional therapy [20]. Overall, 12 papers and a total of 319 patients were included in this review (Table 1). Reperforation after sealing the oroantral fistula was reported in 12 patients, and the overall success rate was 96.2%. The reperforation of oroantral fistula can be caused by the remaining infected tissue in the fistula area [21]. Complete removal of inflammatory tissue is an essential step for a successful operation [18]. As the vascular pedicle of the BFP is fragile, careless handling of the tissue can damage the vascular supply of the flap [18]. Other causes of failure include surgery by an inexperienced surgeon and invasive surgery [22].

**Table 1:** Oroantral fistula treated by BFP

Number of patients	Average age (range)	Re-perforation	Ref
25	45 (35–56)	0	[5]
24	NA	0	[6]
130	39 (15–90)	9	[18]
15	37 (22–57)	1	[19]
7	33 (NA)	0	[20]
14	38 (21–56)	1	[21]
10	38 (NA)	0	[22]
9	51 (29–64)	1	[61]
56	NA (19–56)	0	[62]
12	40 (NA)	0	[63]
11	43 (24–62)	0	[64]
6	44 (32–51)	0	[65]

*NA* not available, *Ref* reference number

Although BFP showed high success rates in sealing oroantral fistula, it could not increase bone regeneration [23]. Therefore, dental implant installation into the reconstructed defect by BFP is not recommended. For bone regeneration, BFP should be used with proper bone graft materials.

### Congenital defect

Cleft palate is a common congenital deformity [24, 25]. It is caused by incomplete fusion of the maxillary process during the developmental stage. Although many etiologic factors such as genetics and the environment have been suggested, the pathogenesis of cleft palate remains controversial [3, 4]. For the treatment of cleft palate, sealing the communication between the oral cavity and the nasal cavity is essential for successful treatment [25, 26]. Many types of flap design have been introduced for the treatment of cleft palate.

The success rate of cleft palate surgery is influenced by many factors. The main flap for the cleft palate surgery is fed by the greater palatine artery and the lesser palatine artery [24]. If the palatal defect is wide, the flap width will be narrowed. These long narrow flaps may cause problems with blood circulation, and wide expanses of exposed raw bone surface can cause extensive wound contracture after the operation [24–26]. Ischemic damage and wound contracture is the main cause of postoperative palatal fistula. The incidence of postoperative palatal fistula is reported to be 4.6–12.5% and is dependent on the degree of the tissue defect [27]. Wound contracture after operation can cause shortening of the soft palate and may result in velopharyngeal insufficiency [28].

**Fig. 4:**
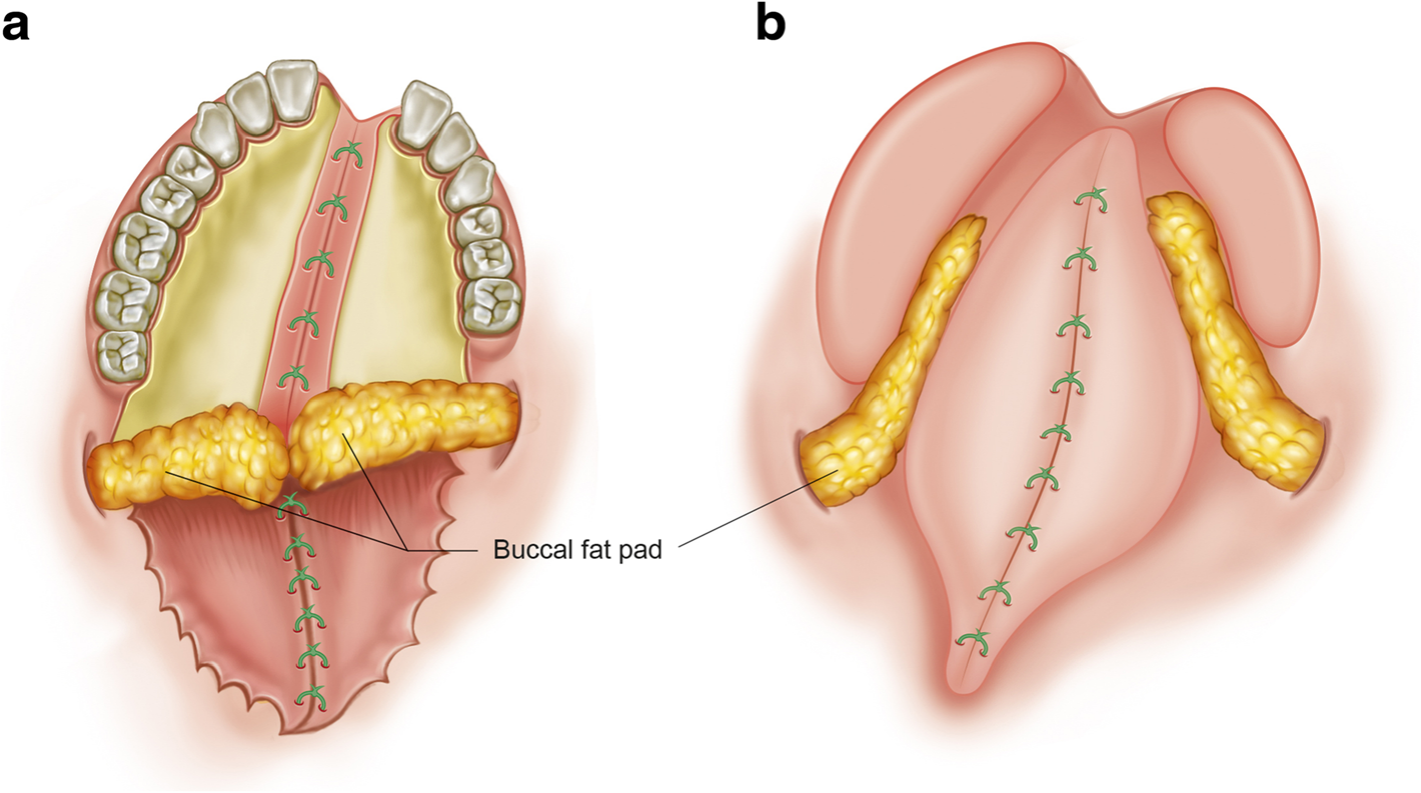
The application of the buccal fat pad flap (BFP) for the treatment of cleft palate. **a** BFP can be placed on the junction between the hard palate and soft palate to prevent possible palatal fistula. **b** BFP can be used for covering the raw bone surface after sealing the palatal flap

The BFP has abundant vascular supply. The operation field for the flap generation is also adjacent to the operation field of cleft palate surgery (Fig. 4). Most cleft palate patients are children, and the BFP is particularly well developed in children. BFP can be placed on the junction between the hard palate and soft palate to prevent possible palatal fistula (Fig. 4a) [24, 25], or it can be used for covering the raw bone surface after sealing the palatal flap (Fig. 4b) [26]. BFP is particularly useful for the repair of secondary defect after cleft palate surgery [3, 4].

Few publications, other than case or technical reports, have discussed BFP’s usefulness for cleft palate surgery. Studies with less than five patients were excluded, and six papers encompassing a total of 101 patients were included (Table 2). Two of the included papers had some common data [3, 4]; therefore, the actual number of patients may be overestimated. Only a single case of postoperative fistula was reported, and it was spontaneously healed without further treatment [3]. A maximum of a 20 × 10-mm palatal defect could be covered with BFP [3, 4]. Tongue flap or temporal fascia flap has been used to repair secondary palatal defects. However, both techniques require extensive operation time and have moderate donor site morbidity. Considering the ease of this technique and availability of BFP, it can be considered for the secondary repair of palatal fistula located at the posterior palate [3, 4]. However, with the current BFP technique, it is difficult to cover defects located at the anterior palate [4].

**Table 2:** Cleft lip and alveolus treated by BFP

Number of patients	Average age (range)	Primary/secondary	Post-operative fistula	Ref
29	NA (2.5–19)	Secondary	1	[3]
20	8.9 (2.5–19)	Secondary	0	[4]
6	7.2 (2–30)	NA	0	[65]
8	28 (19–46 months)	Primary	0	[24]
14	3.2 (11–15 months)	Mixed	0	[25]
24	4.7 (6–17 months)	Mixed	0	[29]

*NA* not available, *Ref* reference number

BFP may be used for the prevention of palatal fistula during palatoplasty [29]. It has been claimed that there is no impairment in function and growth of the palate covered with BFP compared to the use of conventional techniques [29]. However, there has been no comparative study in the function and growth of the palate after pedicled buccal fat pad application. Comparative analysis with conventional technique should be performed to validate the BFP as a preventive measure for cleft palate surgery.

### Osteonecrosis of the jaw bone

Osteonecrosis can result from radiation therapy during the treatment of malignancy [30] or medications, such as bisphosphonate and denosumab [9]. The main mechanism of osteonecrosis is vascular impairment and resultant hypoxia. Additional microbial invasion and dental procedures are subsequent events that lead to the progression of osteonecrosis [31]. Nonsurgical therapy for osteonecrosis consists of regular dressing and prescription of supplemental antibiotics. Because of the avascular nature of the disease, hyperbaric oxygen therapy has also been used in some studies [32]. Surgical intervention involves the complete removal of necrotic bone and subsequent reconstruction with rich vascularized tissue [30]. Microvascular reconstruction has been used for the reconstruction of osteonecrosis because of poorly vascularized tissue beds in recipient sites [33].

After excluding the papers with a small sample size (≤5), only three papers discussing osteonecrosis of the jaw bone were included in this review (Table 3). The total number of patients was 43, and 38 patients showed uneventful healing (88.4%). Two cases of 100% uneventful healing were reported, in which patients showed bone exposure during follow-up after restarting medication [9]. Unsuccessful epithelial healing on the bone is frequently observed in cases with incomplete resection of the necrotic bone [30, 34]. These cases could be treated by additional resection of sequestrum [30, 34].

**Table 3:** Osteonecrosis treated by BFP

Number of patients	Average age (range)	Cause	Location	Uneventful healing	Ref
23	68 (39–93)	Medication	Mx: 23	23	[9]
10	56 (24–74)	Radiation	Mx: 2, Mn: 8	6	[30]
10	73 (57–81)	Medication	Mx: 2, Mn: 8	9	[34]

*Mx* maxilla, *Mn* mandible, *Ref* reference number

Some cases of osteonecrosis are poorly responsive to conservative therapy [30, 34]. For example, the success rate of conservative therapy for osteoradionecrosis has been reported to be 37–44% [35, 36]. Patients who cannot be treated by conservative therapy should receive surgical intervention. The location and size of the osteonecrosis are sometimes an obstacle for reconstruction with BFP [30]. Lower reconstructive success rates have been found with advanced stages of osteoradionecrosis compared to early stages [30]. A microvascular free flap can be used for advanced stages of osteoradionecrosis [33].

### Cyst or tumor

**Fig. 5:**
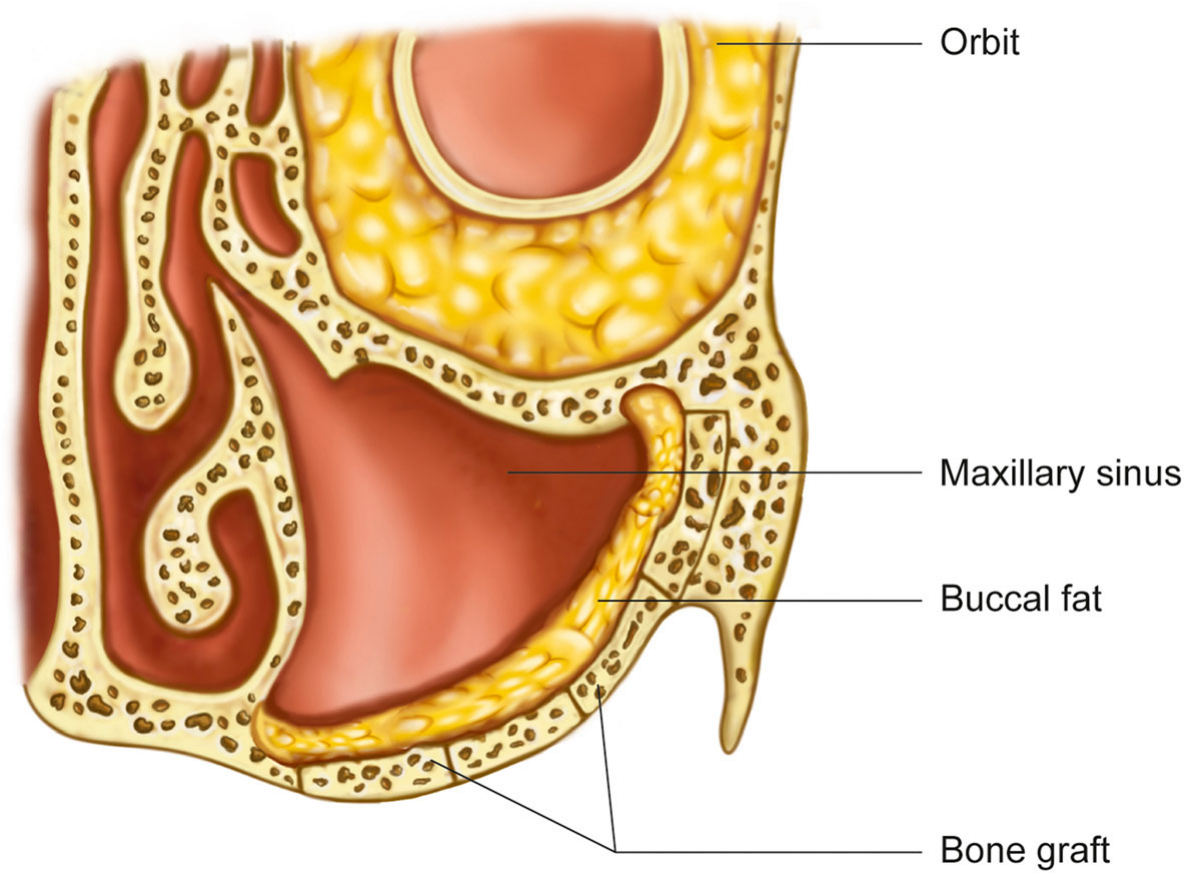
The application of the buccal fat pad flap (BFP) after tumor resection. BFP can be used with free bone graft for the reconstruction of the maxillary sinus wall

BFP has been frequently used in the successful reconstruction of intraoral defects, including those induced by benign tumors and cysts (Fig. 5). The first clinical application of BFP was for the closure of a defect in the palate induced by a tumor [13]. In Egyedi’s report [13], a split-thickness skin graft was applied on the BFP. Later, Tideman et al. [37] reported that epithelialization could be observed on the BFP without skin graft. The regenerated epithelium is parakeratinized stratified squamous epithelium and looks similar to the adjacent oral epithelium [38]. In cases of moderate-sized palatal defects, BFP allows early epithelialization without postoperative discomfort [39].

Twelve papers were included in this review after excluding the papers with a small sample size (≤5) (Table 4). The total number of patients was 202, and 180 patients showed uneventful healing (89.1%). Eighty-six defects were induced by malignant tumor, and 102 were induced by benign tumors. The anatomic location of the defect was mainly the maxilla (*n* = 141). Posterior mandible or buccal mucosal defects can also be restored by BFP, but a tumor-free resection margin is essential for successful treatment [40].

**Table 4:** Cyst or tumor treated by BFP

Number of patients	Average age (range)	Cause	Location	Uneventful healing	Complication	Ref
22	67.5 (26–83)	Mal: 12, Be: 10	Mx: 14, Mn: 2, B: 4, FOM: 1, TMJ: 1	22	0	[7]
15	27.9 (17–50)	Be: 15	Mx: 15	13	Inf: 2	[8]
11	34.4 (15–60)	Be: 5, C: 6	Mx: 11	8	GL:2, Bl: 1	[19]
11	57.6 (42–70)	Mal: 7, Be: 4	Mx: 10, B: 1	11	0	[65]
12	60.6 (32–90)	Mal: 10, Be: 1, C: 1	Mx: 4, Mn: 7, B: 1	11	Inf: 1	[37]
28	52 (9–85)	Mal: 19, Be: 8, C: 1	Mx: 22, Mn: 2, B: 1, Mix: 3	28	0	[38]
6	54.7 (41–69)	Mal: 3, Be: 3	Mx: 6	5	GL: 1	[39]
15	57.9 (34–78)	Mal: 10, Be: 5	Mx: 5, Mn: 3, B: 7	8	MOL: 7	[43]
15	NA	Mal: 15	Mx: 6, Mn: 3, B: 6	13	GL: 2	[45]
38	26 (14–54)	Be: 36, C: 2	Mx: 38	35	Fistula: 3	[41]
21	NA (28–72)	Mal: 10, Be: 11	Mx: 2, Mn: 2, B: 16, Mix: 1	20	Tumor invasion: 1	[40]
8	36.6 (20–68)	Be: 4, C: 4	Mx: 8	6	Fistula: 1, Deh: 1	[42]

*Ref* reference number, *Mal* malignancy, *Be* benign, *C* cyst, *Mx* maxilla, *Mn* mandible, *B* buccal mucosa, *MOL* mouth opening limitation, *NA* not available, *GL* loss of graft, *Inf* infection, *Bl* bleeding, *Deh* dehiscence

If there is a sound oral epithelium, BFP can be used for the coverage of autogenous free block bone graft [41, 42] or titanium mesh with particulate bone [8] on the opposite side of the sinus. As the maxillary sinus mucosa is thin and frequently removed during tumor surgery, well-vascularized BFP can be substituted for sinus mucosa to cover a bone graft [42]. When the BFP is used as a barrier for free bone graft, the incidence of infection and graft resorption may be reduced [41].

When using BFP to treat defects induced by a malignant tumor, postoperative radiation therapy should be considered. Any supplementary cancer therapy can induce bone exposure and fistula [38, 40]. As BFP can be used for the repair of bone necrosis defect, it should be spared for future use in malignant tumor patients [30]. For the reconstruction of tumor defects, excessive fat is required at times, and the patient may show limitation of mouth opening [43]. As the function of the buccal fat pad is lubrication during contracture of multiple muscles [44], loss of buccal fat can induce scar contracture and adhesion of muscles [43]. Therefore, active mouth-opening exercise is advised for these patients [43]. Excessive graft taking may also induce cheek depression [40].

Although there have been many successful applications of BFP for the reconstruction of tumors, the method also has limitations. Defect sizes exceeding 4 cm × 4 cm × 3 cm have higher failure rates [45]. Other authors have also advised that it should not be used for defects larger than 6 cm × 4 cm [8]. In a previous review, the complication rate was 16.4% among 165 cases of BFP graft [45]. The most frequent complication was breakdown followed by postoperative fistula formation [45].

Oral submucous fibrosis is a precancerous lesion in the oral mucosa. Mouth opening limitation due to fibrous contracture is a major clinical feature. Abnormal sensation of oral mucosa is also an accompanying symptom of oral submucous fibrosis [46]. As chewing areca nut is reported to be a potential etiology, oral submucous fibrosis is prevalent in India [46–49] and Taiwan [50]. Complete cure for oral submucous fibrosis has barely been achieved [50]. Accordingly, functional restoration has been the main goal of the treatment.

There have been several reports about the application of BFP for the treatment of oral submucous fibrosis. Five papers were included in this review after excluding the papers with small sample sizes (≤5) (Table 5). Although the results have been described as favorable, evaluation criteria are unclear in most papers. The most important evaluation criteria for the treatment of oral submucous fibrosis should be long-term stability of mouth function. When BFP graft was compared to other surgical protocols, such as tongue flap, nasolabial flap, and free skin graft, there was no difference in mouth-opening ability during follow-up [46]. The exact demographic data such as patient’s age, potential etiology, the size of the lesion, and postoperative follow-up should be provided in future reports. As limitation of mouth opening has been reported as a complication of BFP [43], detailed surgical protocol about the graft amount should also be suggested. The overall evidence of BFP application in oral submucous fibrosis seems insufficient.

**Table 5:** Oral submucous fibrosis treated by BFP

Number of patients	Average age (range)	Uneventful healing	Compl	Ref
25	34 (17–54)	25	0	[46]
28	NA (18–53)	28	0	[47]
10	NA	NA	NA	[48]
20	NA	19	MOL: 1	[49]
16	NA (20–22)	NA	NA	[50]

*Compl* complications, *Ref* reference number, *NA* not available, *MOL* mouth opening limitation

### Other applications

BFP has also been used as an interpositioning material for temporomandibular joint reconstruction. Free fat graft from the abdomen is used for the reconstruction of the temporomandibular joint, which results in functional improvement [51]. In contrast to the abdominal fat, BFP can be used as a pedicled flap because of its anatomical proximity [52]. When BFP is used for gap arthroplasty of the temporomandibular joint, minimal gap (6–7 mm) is advised [53]. The shrinkage rate is reported to be 28% [53]. If the prepared gap is large, a greater amount of fat is required, and vertical height of the mandibular ramus cannot be maintained [54]. Compared to temporal fascia graft, BFP is resilient and does not have muscle [55]. BFP is stable after grafting into the temporomandibular joint and can be detected on MRI 1 year after the operation [56].

In patients receiving maxillary advancement surgery by LeFort I osteotomy, the upper lip usually loses its normal concavity [57]. To improve the upper lip profile, BFP can be used as an augmentation material [57]. Skull base defect after tumor surgery also can be repaired by BFP [58]. After parotid gland resection, BFP can be used for the prevention of Frey syndrome [59]. BFP is also used for repairing perforated maxillary sinus membrane during dental implant surgery [60].

## Conclusions

Since the introduction of BFP for the reconstruction of the maxilla [13], many applications have been introduced. BFP has many advantages over other types of flaps. The surgical procedure is simple and has shown a high success rate in various applications. BFP can be used in epithelialization without additional skin graft. The rich vascularity of BFP is an advantage when it is used in a poorly vascularized recipient site. However, its size is a limitation, and repeated usage may not be possible. As the flap is fragile, damage to the vascular pedicle may result in graft loss. Removal of too much of the buccal fat pad may induce facial disfigurement or mouth opening limitation. These limitations should be considered for the clinical application of BFP.

## Abbreviation

BFP: Buccal fat pad flap

## References

[1] Soutar DS, Scheker LR, Tanner NSB, McGregor IA (1983). The radial forearm flap: a versatile method for intra-oral reconstruction. Br J Plast Surg.

[2] Sadig W, Almas K (2004). Risk factors and management of dehiscent wounds in implant dentistry. Implant Dent.

[3] Ashtiani AK, Bohluli B, Kalantar Motamedi MH, Fatemi MJ, Moharamnejad N (2011). Effectiveness of buccal fat in closing residual midpalatal and posterior palatal fistulas in patients previously treated for clefts. J Oral Maxillofac Surg.

[4] Ashtiani AK, Fatemi MJ, Pooli AH, Habibi M (2011). Closure of palatal fistula with buccal fat pad flap. Int J Oral Maxillofac Surg.

[5] Daif ET (2016). Long-term effectiveness of the pedicled buccal fat pad in the closure of a large oroantral fistula. J Oral Maxillofac Surg.

[6] Adams T, Taub D, Rosen M (2015). Repair of oroantral communications by use of a combined surgical approach: functional endoscopic surgery and buccal advancement flap/buccal fat pad graft. J Oral Maxillofac Surg.

[7] Toshihiro Y, Nariai Y, Takamura Y, Yoshimura H, Tobita T, Yoshino A (2013). Applicability of buccal fat pad grafting for oral reconstruction. Int J Oral Maxillofac Surg.

[8] Liu YM, Chen GF, Yan JL, Zhao SF, Zhang WM, Zhao S (2006). Functional reconstruction of maxilla with BFP, prefabricated titanium mesh and autologous bone grafts. Int J Oral Maxillofac Surg.

[9] Melville JC, Tursun R, Shum JW, Young S, Hanna IA, Marx RE (2016). A technique for the treatment of oral-antral fistulas resulting from medication-related osteonecrosis of the maxilla: the combined buccal fat pad flap and radical sinusotomy. Oral Surg Oral Med Oral Pathol Oral Radiol.

[10] El Deeb M, Roszkowski M, El Hakim I (1990). Tissue response to hydroxylapatite in induced diabetic and nondiabetic rats: histologic evaluation. J Oral Maxillofac Surg.

[11] Drake DB, Oishi SN (1995). Wound healing considerations in chemotherapy and radiation therapy. Clin Plast Surg.

[12] Colen SR, Shaw WW, McCarthy JG (1986). Review of the morbidity of 300 free-flap donor sites. Plast Reconstr Surg.

[13] Egyedi P (1977). Utilization of the buccal fat pad for closure of oro-antral and/or oro-nasal communications. J Maxillofac Surg.

[14] Arce K (2007). Buccal fat pad in maxillary reconstruction. Atlas Oral Maxillofac Surg Clin North Am.

[15] Singh J, Prasad K, Lalitha RM, Ranganath K (2010). Buccal pad of fat and its applications in oral and maxillofacial surgery: a review of published literature (February) 2004 to (July) 2009. Oral Surg Oral Med Oral Pathol Oral Radiol Endod.

[16] Tostevin PM, Ellis H (1995). The buccal pad of fat: a review. Clin Anat.

[17] Stuzln JM, Wagstrom L, Kawamoto HK, Baker TJ, Wolfe SA (1990). The anatomy and clinical applications of the buccal fat pad. Plast Reconstr Surg.

[18] Poeschl PW, Baumann A, Russmueller G, Poeschl E, Klug C, Ewers R (2009). Closure of oroantral communications with Bichat’s buccal fat pad. J Oral Maxillofac Surg.

[19] Alkan A, Dolanmaz D, Uzun E, Erdem E (2003). The reconstruction of oral defects with buccal fat pad. Swiss Med Wkly.

[20] Abad-Gallegos M, Figueiredo R, Rodríguez-Baeza A, Gay-Escoda C (2011). Use of Bichat’s buccal fat pad for the sealing of orosinusal communications. A presentation of 8 cases. Med Oral Patol Oral Cir Bucal.

[21] Hanazawa Y, Itoh K, Mabashi T, Sato K (1995). Closure of oroantral communications using a pedicled buccal fat pad graft. J Oral Maxillofac Surg.

[22] Nezafati S, Vafaii A, Ghojazadeh M (2012). Comparison of pedicled buccal fat pad flap with buccal flap for closure of oro-antral communication. Int J Oral Maxillofac Surg.

[23] Hariram, Pal US, Mohammad S, Singh RK, Singh G, Malkunje LR (2010). Buccal fat pad versus sandwich graft for treatment of oroantral defects: a comparison. Natl J Maxillofac Surg.

[24] Zhang Q, Li L, Tan W, Chen L, Gao N, Bao C (2010). Application of unilateral pedicled buccal fat pad for nasal membrane closure in the bilateral complete cleft palate. J Oral Maxillofac Surg.

[25] Levi B, Kasten SJ, Buchman SR (2009). Utilization of the buccal fat pad flap for congenital cleft palate repair. Plast Reconstr Surg.

[26] Kim YK (2001). The use of a pedicled buccal fat pad graft for bone coverage in primary palatorrhaphy: a case report. J Oral Maxillofac Surg.

[27] Musgrave RH, Bremner JC (1960). Complications of cleft palate surgery. Plast Reconstr Surg Transplant Bull.

[28] Khouw YL, van der Wal KG, Bartels F, van der Biezen JJ (2004). Bilateral palatal reconstruction using 2 pedicled buccal fat pads in rhinolalea aperta after extensive necrotizing tonsillitis: a case report. J Oral Maxillofac Surg.

[29] Gröbe A, Eichhorn W, Hanken H, Precht C, Schmelzle R, Heiland M (2011). The use of buccal fat pad (BFP) as a pedicled graft in cleft palate surgery. Int J Oral Maxillofac Surg.

[30] Nabil S, Ramli R (2012). The use of buccal fat pad flap in the treatment of osteoradionecrosis. Int J Oral Maxillofac Surg.

[31] Marx RE (2009). Reconstruction of defects caused by bisphosphonate-induced osteonecrosis of the jaws. J Oral Maxillofac Surg.

[32] Freiberger JJ (2009). Utility of hyperbaric oxygen in treatment of bisphosphonate-related osteonecrosis of the jaws. J Oral Maxillofac Surg.

[33] Chang DW, Oh HK, Robb GL, Miller MJ (2001). Management of advanced mandibular osteoradionecrosis with free flap reconstruction. Head Neck.

[34] Rotaru H, Kim MK, Kim SG, Park YW (2015). Pedicled buccal fat pad flap as a reliable surgical strategy for the treatment of medication-related osteonecrosis of the jaw. J Oral Maxillofac Surg.

[35] Wong JK, Wood RE, McLean M (1997). Conservative management of osteoradionecrosis. Oral Surg Oral Med Oral Pathol Oral Radiol Endod.

[36] Beumer J, Harrison R, Sanders B, Kurrasch M (1984). Osteoradionecrosis: predisposing factors and outcomes of therapy. Head Neck Surg.

[37] Tideman H, Bosanquet A, Scott J (1986). Use of the buccal fat pad as a pedicled graft. J Oral Maxillofac Surg.

[38] Samman N, Cheung LK, Tideman H (1993). The buccal fat pad in oral reconstruction. Int J Oral Maxillofac Surg.

[39] Fujimura N, Nagura H, Enomoto S (1990). Grafting of the buccal fat pad into palatal defects. J Craniomaxillofac Surg.

[40] Hao SP (2000). Reconstruction of oral defects with the pedicled buccal fat pad flap. Otolaryngol Head Neck Surg.

[41] Zhong LP, Chen GF, Fan LJ, Zhao SF (2004). Immediate reconstruction of maxilla with bone grafts supported by pedicled buccal fat pad graft. Oral Surg Oral Med Oral Pathol Oral Radiol Endod.

[42] Vuillemin T, Raveh J, Ramon Y (1988). Reconstruction of the maxilla with bone grafts supported by the buccal fat pad. J Oral Maxillofac Surg.

[43] Colella G, Tartaro G, Giudice A (2004). The buccal fat pad in oral reconstruction. Br J Plast Surg.

[44] Yousuf S, Tubbs RS, Wartmann CT, Kapos T, Cohen-Gadol AA, Loukas M (2010). A review of the gross anatomy, functions, pathology, and clinical uses of the buccal fat pad. Surg Radiol Anat.

[45] Rapidis AD, Alexandridis CA, Eleftheriadis E, Angelopoulos AP (2000). The use of the buccal fat pad for reconstruction of oral defects: review of the literature and report of 15 cases. J Oral Maxillofac Surg.

[46] Mehrotra D, Pradhan R, Gupta S (2009). Retrospective comparison of surgical treatment modalities in 100 patients with oral submucous fibrosis. Oral Surg Oral Med Oral Pathol Oral Radiol Endod.

[47] Sharma R, Thapliyal GK, Sinha R, Menon PS (2012). Use of buccal fat pad for treatment of oral submucous fibrosis. J Oral Maxillofac Surg.

[48] Rai A, Datarkar A, Rai M (2014). Is buccal fat pad a better option than nasolabial flap for reconstruction of intraoral defects after surgical release of fibrous bands in patients with oral submucous fibrosis? A pilot study: a protocol for the management of oral submucous fibrosis. J Craniomaxillofac Surg.

[49] Lambade P, Dawane P, Thorat A (2016). Efficacy of buccal fat pad in the surgical management of oral submucous fibrosis: a prospective study. Oral Maxillofac Surg.

[50] Chao CK, Chang LC, Liu SY, Wang JJ (2002). Histologic examination of pedicled buccal fat pad graft in oral submucous fibrosis. J Oral Maxillofac Surg.

[51] Wolford LM, Karras SC (1997). Autologous fat transplantation around temporomandibular joint total joint prostheses: preliminary treatment outcomes. J Oral Maxillofac Surg.

[52] Rattan V (2006). A simple technique for use of buccal pad of fat in temporomandibular joint reconstruction. J Oral Maxillofac Surg.

[53] Bansal V, Bansal A, Mowar A, Gupta S (2015). Ultrasonography for the volumetric analysis of the buccal fat pad as an interposition material for the management of ankylosis of the temporomandibular joint in adolescent patients. Br J Oral Maxillofac Surg.

[54] Singh V, Dhingra R, Sharma B, Bhagol A, Kumar P (2011). Retrospective analysis of use of buccal fat pad as an interpositional graft in temporomandibular joint ankylosis: preliminary study. J Oral Maxillofac Surg.

[55] Singh V, Dhingra R, Bhagol A (2012). Prospective analysis of temporomandibular joint reconstruction in ankylosis with sternoclavicular graft and buccal fat pad lining. J Oral Maxillofac Surg.

[56] Gaba S, Sharma RK, Rattan V, Khandelwal N (2012). The long-term fate of pedicled buccal pad fat used for interpositional arthroplasty in TMJ ankylosis. J Plast Reconstr Aesthet Surg.

[57] Rubio-Bueno P, Ardanza B, Piñas L, Murillo N (2013). Pedicled buccal fat pad flap for upper lip augmentation in orthognathic surgery patients. J Oral Maxillofac Surg.

[58] Cherekaev VA, Golbin DA, Belov AI (2012). Translocated pedicled buccal fat pad: closure of anterior and middle skull base defects after tumor resection. J Craniofac Surg.

[59] Kim JT, Naidu S, Kim YH (2010). The buccal fat: a convenient and effective autologous option to prevent Frey syndrome and for facial contouring following parotidectomy. Plast Reconstr Surg.

[60] Kim YK, Yun PY, Oh JS, Kim SG (2014). Prognosis of closure of large sinus membrane perforations using pedicled buccal fat pads and a resorbable collagen membrane: case series study. J Korean Assoc Oral Maxillofac Surg.

[61] Alonso-González R, Peñarrocha-Diago M, Peñarrocha-Oltra D, Aloy-Prósper A, Camacho-Alonso F, Peñarrocha-Diago M (2015). Closure of oroantral communications with Bichat’s buccal fat pad. Level of patient satisfaction. J Clin Exp Dent.

[62] Stajcić Z (1992). The buccal fat pad in the closure of oro-antral communications: a study of 56 cases. J Craniomaxillofac Surg.

[63] Baumann A, Ewers R (2000). Application of the buccal fat pad in oral reconstruction. J Oral Maxillofac Surg.

[64] Jain MK, Ramesh C, Sankar K, Lokesh Babu KT (2012). Pedicled buccal fat pad in the management of oroantral fistula: a clinical study of 15 cases. Int J Oral Maxillofac Surg.

[65] Chaudhary B, Gong Z, Lin Z, Abbas K, Ling B, Liu H (2014). Reconstruction of intraoral maxillary defect with buccal fat pad. J Craniofac Surg.

